# Cytotoxic Profiling of 3′,4′,5′‐Trimethoxychalcones Reveals Cell‐Line‐Dependent Cytotoxic Activity: An In Vitro and In Silico Study

**DOI:** 10.1002/cbdv.71336

**Published:** 2026-05-13

**Authors:** Aleksandar Y. Mehandzhiyski, Abdessamad Beraich, Zhivko Velkov, Maya Zaharieva, Spiro Konstantinov, Daniela Batovska

**Affiliations:** ^1^ Laboratory of Organic Electronics Department of Science and Technology (ITN) Linköping University Norrköping Sweden; ^2^ Physical Chemistry of the Natural Resources and Processes, Department of Chemistry, Faculty of Sciences Mohamed First University Oujda Morocco; ^3^ Department of Chemistry South‐West University “Neofit Rilski” Blagoevgrad Bulgaria; ^4^ Department of Infectious Microbiology The Stephan Angeloff Institute of Microbiology Bulgarian Academy of Sciences Sofia Bulgaria; ^5^ Department of Pharmacology, Pharmacotherapy and Toxicology Medical University of Sofia, Faculty of Pharmacy Sofia Bulgaria; ^6^ Institute of Chemical Engineering Bulgarian Academy of Sciences Sofia Bulgaria

**Keywords:** cell line–dependent activity, chalcones, cytotoxicity, molecular docking

## Abstract

Cytotoxic activity of a series of 3′,4′,5′‐trimethoxychalcones (**1–25**) was evaluated against multiple human cancer cell lines under uniform experimental conditions. The compounds were designed to bear simple substitutions on ring B to allow direct comparison of substitution effects. Most derivatives showed higher cytotoxic potency than the reference drug melphalan in the more responsive models. Several compounds exhibited submicromolar half‐maximal inhibitory concentration (IC_50_) values (**7**, **8**, **12**, **13**, **14**, **17**, **18**, **20**, **23**). Low‐nanomolar activity was also observed. Compounds **4**, **5**, **9**, **10**, **15**, **16**, and **24** showed particularly high potency. However, cytotoxic responses were strongly cell‐line dependent. MCF‐7 (estrogen receptor–positive breast carcinoma) and SKW‐3 (T‐cell leukemia) cells were highly sensitive, whereas MDA‐MB‐231 (triple‐negative breast carcinoma) and K‐562 (BCR‐ABL–positive leukemia) cells were less responsive. In the doxorubicin‐resistant HL‐60/DOX (multidrug‐resistant promyelocytic leukemia) subline, **10** retained activity. Compound **16** showed a nine‐fold increase in potency relative to HL‐60. Hierarchical clustering, principal component analysis (PCA), and descriptor‐based quantitative structure–activity relationship (QSAR) revealed no transferable trends. Molecular docking showed moderate, non‐selective interactions with multiple targets. Similar binding to human serum albumin was observed. Overall, these findings support cell‐line‐resolved evaluation and context‐aware optimization in 3′,4′,5′‐trimethoxychalcone‐based anticancer research.

## Graphical abstract

A systematic evaluation of 3′,4′,5′‐trimethoxychalcones reveals potent yet strongly cell‐line‐dependent cytotoxic activity across multiple cancer models. Despite high potency, no universal structure–activity relationships emerge. Combined in vitro and in silico analyses indicate a multifactorial, context‐dependent mode of action, underscoring the need for cell‐specific evaluation in chalcone‐based anticancer drug design.

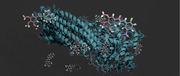

**Abbreviations**
HCA: hierarchical clustering analysis
PCA: principal component analysis
SAR: structure–activity relationship
QSAR: quantitative structure‐activity relationship
HSA: human serum albumin

## 1 Introduction

Chalcones are α,β‐unsaturated ketones in which two aromatic rings are connected through an enone bridge, giving rise to a structurally simple yet chemically versatile scaffold of long‐standing interest in medicinal and bioorganic chemistry. Their straightforward synthesis and amenability to substitution have positioned chalcones as a widely used platform for the exploration of biologically active small molecules, including anticancer agents [1, 2, 3, 4]. These properties also facilitate systematic structure–activity relationship (SAR) analyses through controlled modification of substitution patterns while preserving the core scaffold.

Within the chalcone family, derivatives bearing a 3′,4′,5′‐trimethoxyphenyl moiety have repeatedly been associated with cytotoxic and antiproliferative effects. Such effects have been reported across multiple cancer cell lines, supporting the view of this substitution pattern as a privileged pharmacophore [5, 6, 7, 8]. Reported molecular interactions for trimethoxy‐substituted chalcones include modulation of tubulin dynamics, transcription‐related signaling pathways, apoptosis‐associated proteins, and drug‐efflux mechanisms [5, 8, 9, 10]. However, the diversity of proposed targets and experimental models suggests that their cytotoxic effects do not arise from a single universal mechanism but rather reflect context‐dependent interactions influenced by both molecular structure and cellular background.

A central theme in SAR analyses of 3′,4′,5′‐trimethoxychalcones is the influence of structural variation on the second aromatic ring (ring B). Numerous studies report a pronounced impact of ring‐B substitution on cytotoxic potency, with *para*‐substituted electron‐withdrawing and moderately lipophilic groups—such as halogens or nitro functionalities—often identified as favorable within individual compound series [11, 12]. At the same time, substantial modulation of activity has also been observed for electron‐donating substituents (e.g., hydroxy or methoxy groups), as well as for bulkier or more polar functionalities. Importantly, these trends emerge from chemically and methodologically heterogeneous datasets, in which differences in substitution strategy, library size, assay design, treatment duration, and cancer cell models limit direct comparability across studies [5, 13, 14, 15]. These limitations underscore the need for comparative studies employing defined compound libraries evaluated under uniform experimental conditions.

In parallel with efforts to refine substituent‐driven SARs, a clear trend has emerged toward increasing structural elaboration of chalcone derivatives. Numerous studies describe analogues incorporating heterocyclic units, fused aromatic systems, extended side chains, or multifunctional polar groups, typically with the aim of enhancing potency or target selectivity [2, 15, 16, 17, 18, 19, 20]. While such strategies expand chemical space and have produced compounds with encouraging in vitro activity, they also introduce greater molecular complexity and may adversely affect key physicochemical properties, including solubility, membrane permeability, and bioavailability [15]. Consequently, interpretation of cytotoxicity data becomes more challenging, and the degree to which observed effects can be attributed to defined structural features of the classical chalcone scaffold is often reduced.

As a result, much of the literature on chalcone‐based anticancer agents is characterized either by screening‐oriented studies confined to a single cancer cell model [5, 8, 12, 21] or by focused investigations of small compound sets evaluated under narrowly defined experimental conditions [11, 13, 14, 22]. While both approaches have yielded valuable insights, they often provide limited evidence for the robustness of apparent substitution effects when examined across different cellular systems under consistent methodological frameworks [1, 2, 22]. This gap underscores the need for comparative studies that deliberately limit chemical complexity while applying uniform experimental conditions across multiple cancer models [1, 2].

Against this background, the present study aims to examine the cytotoxic activity of a defined library of 25 3′,4′,5′‐trimethoxychalcones. Systematically varied substitutions on the second aromatic ring enable direct comparison of their effects within a controlled structural framework. Cytotoxic activity is evaluated across multiple human cancer cell lines under uniform experimental conditions. Another goal is to assess whether widely used analytical approaches can adequately capture structure–activity relationships. This design distinguishes the present study from previous smaller sets of trimethoxychalcones evaluated in limited cell models under heterogeneous conditions.

## 2 Materials and Methods

### 2.1 Chemicals and Reagents

All solvents and reagents used in this study were of analytical or cell culture grade. RPMI‐1640 culture medium, fetal calf serum (FCS), L‐glutamine, nonessential amino acids, sodium pyruvate, human insulin, and phosphate‐buffered saline (PBS) were obtained from Gibco (Thermo Fisher Scientific, Waltham, MA, USA). The tetrazole salt MTT (3‐(4,5‐dimethylthiazol‐2‐yl)‐2,5‐diphenyltetrazolium bromide) and melphalan were purchased from Sigma‐Aldrich (St. Louis, MO, USA).

The chalcones investigated in this study belong to a well‐established 3′,4′,5′‐trimethoxychalcone scaffold. They were obtained from a library of *trans*‐chalcones synthesized and fully characterized in earlier studies, following reported procedures [6, 7, 23, 24, 25]. Their identity was consistent with reported data, and no impurities were detected in the NMR spectra. All compounds were obtained as trans‐isomers, as indicated by the characteristic coupling constant (*J* ≈ 15.5 Hz).^1^H and ^13^C NMR data for all 25 compounds are presented in the Supplementary Information.

### 2.2 Cell Lines and Culture Conditions

The cytotoxic activity of the investigated chalcones was evaluated in vitro against a panel of human cancer cell lines obtained from the German Collection of Microorganisms and Cell Cultures (DSMZ, Braunschweig, Germany). The panel comprised MDA‐MB‐231 (estrogen receptor–negative, ER^−^, breast carcinoma, RRID:CVCL_0062), MCF‐7 (estrogen receptor–positive, ER^+^, breast carcinoma, RRID:CVCL_0031), SKW‐3 (syn. KE‐37; T‐cell leukemia, RRID:CVCL_2197), and K‐562 (erythroleukemia, BCR‐ABL–positive, Philadelphia chromosome–positive, RRID:CVCL_0004). In addition, a subset of chalcones was evaluated against the human promyelocytic leukemia cell line HL‐60 (RRID:CVCL_0002) and its doxorubicin‐resistant subline HL‐60/DOX (RRID:CVCL_0304), which overexpresses the multidrug resistance (MDR)–associated protein MRP‐1. The human bladder carcinoma cell lines SW‐1710 (RRID:CVCL_1721) and Cal‐29 (RRID:CVCL_1808) were included as supplementary models for comparative assessment.

All cell lines were maintained at 37°C in a humidified atmosphere containing 5% CO_2_. MCF‐7 cells were cultured in RPMI‐1640 medium supplemented with 10% fetal calf serum (FCS), nonessential amino acids, 1 mM sodium pyruvate, and 10 µg/mL human insulin. All other cell lines (SKW‐3, K‐562, MDA‐MB‐231, HL‐60, and HL‐60/DOX) were cultured in RPMI‐1640 medium supplemented with 10% FCS and 2 mM L‐glutamine.

### 2.3 In Vitro Cytotoxicity Assay

Cell viability was evaluated using the MTT colorimetric assay in accordance with the principles outlined in ISO 10993‐5:2009 for the in vitro assessment of cytotoxicity, following the method originally described by Mosmann, with minor modifications [26, 27, 28]. Briefly, cells were seeded into 96‐well microplates at densities of 5 × 10^3^–1 × 10^4^ cells per well, depending on the cell line, to ensure exponential growth throughout the assay period. The final volume per well was 100 µL, and cells were allowed to equilibrate prior to treatment. The investigated chalcones were applied at increasing concentrations spanning the sub‐micromolar to 100 µM range and incubated with the cells for 72 h. Stock solutions of each compound were prepared in dimethyl sulfoxide (DMSO) and subsequently diluted with culture medium; the final DMSO concentration in all wells did not exceed 1% (v/v), a level shown to have no effect on cell viability. For each concentration, at least eight technical replicate wells were analyzed within a single experimental run. Control wells contained culture medium with the corresponding concentration of DMSO, and blank wells with MTT but no cells were used for background correction.

Following the treatment period, 10 µL of MTT solution (5 mg/mL in phosphate‐buffered saline) was added to each well, and the plates were incubated for an additional 4 h at 37°C to allow enzymatic reduction of MTT to insoluble formazan crystals by metabolically active cells. The supernatant was then carefully removed, and the resulting formazan crystals were solubilized using 5% formic acid in isopropanol. Absorbance was measured at 570 nm using an ELx800 microplate reader (BioTek Instruments, Shoreline, WA, USA).

After background correction, absorbance values from chalcone‐treated wells were normalized to the control and expressed as percentage cell viability. IC_50_ values were defined as the concentration required to reduce cell viability by 50% and were determined by nonlinear regression of concentration–response curves using GraphPad Prism (version 6.0.0, GraphPad Software, Boston, MA, USA). Results are reported as IC_50_ values with corresponding 95% confidence intervals. Melphalan, a clinically established antineoplastic agent, was used as the reference compound.

### 2.4 Hierarchical Clustering and Heatmap Visualization

Hierarchical clustering analysis (HCA) was applied to examine patterns in cytotoxic activity of chalcone derivatives across the tested cancer cell lines. IC_50_ values were transformed to −log_10_(IC_50_) and subsequently z‐score normalized within each cell line to emphasize relative activity patterns. For data visualization and clustering purposes, IC_50_ values reported as greater than a specified threshold (e.g., > 100 µM) were conservatively assigned the corresponding threshold value prior to −log(IC_50_) transformation. It should be noted that these values are only three out of 100, and they did not affect the cluster analysis. Unsupervised agglomerative clustering was performed for both compounds and cell lines using Ward's linkage method with Euclidean distance as the similarity metric. The resulting clusters were visualized as a heatmap with accompanying dendrograms. All analyses were carried out in Python (version 3.11) using scikit‐learn (version 1.8.0) for clustering and Seaborn (version 0.13.2) for visualization.

### 2.5 Principal Component Analysis (PCA)

Principal component analysis (PCA) was applied to the same pIC_50_ activity matrix to reduce dimensionality and identify dominant patterns in cytotoxicity profiles across cell lines. The first two principal components (PC1 and PC2), capturing the majority of variance in the dataset, were retained and visualized. Compounds were projected into principal component space to reveal global structure–activity relationships and similarity patterns based on biological response profiles. Data points were colored according to unsupervised clustering assignments, and representative chemical structures were annotated for selected compounds exhibiting high and low cytotoxic activity.

### 2.6 QSAR Analysis

QSAR analysis was performed for the investigated 3′,4′,5′‐trimethoxychalcone derivatives to explore associations between molecular properties and cytotoxic activity. Electronic and molecular descriptors were obtained from frontier molecular orbital calculations performed using Gaussian 16 [29], including the energies of the highest occupied molecular orbital (E_HOMO_) and the lowest unoccupied molecular orbital (E_LUMO_). From these values, global reactivity descriptors—electronegativity (χ), chemical hardness (η), softness (S), electrophilicity index (ω), ionization potential (I), electron affinity (A), dipole moment (μ), and chemical potential (ν)—were derived according to standard theoretical definitions [30]. The density functional theory (DFT) calculations were performed utilizing the B3LYP functional and the 6‐311+G(d) basis set. The molecular geometry of all chalcones was optimized, and all electronic descriptors were calculated on the optimized structures.

Experimentally determined IC_50_ values from the in vitro cytotoxicity assays were used as biological response variables. Linear correlation analysis was applied to examine relationships between molecular descriptors and cytotoxic activity across the tested human cancer cell lines.

### 2.7 Molecular Docking

Molecular docking was applied as an exploratory tool to assess whether differential engagement of selected cancer‐relevant proteins could plausibly account for the observed variability in cytotoxic response. The protein targets were selected based on their involvement in apoptosis, multidrug resistance, and intracellular transport. The crystal structures of Bcl‐2 (PDB ID: 2O2F), ABCG2 (BCRP; PDB ID: 6FFC), ABCB1 (P‐gp; PDB ID: 6C0V), the IκBα/NF‐κB complex (PDB ID: 1NFI), and human serum albumin (HSA; PDB IDs: 1SAO and 3GJQ) were retrieved from the Protein Data Bank.

Protein structures were prepared by removing co‐crystallized ligands and crystallographic water molecules, followed by the addition of polar hydrogen atoms and assignment of partial charges using AutoDock Tools (version 1.5.7). Ligand structures were generated and energy‐minimized using Open Babel (version 3.1.1).

Docking simulations were carried out using AutoDock Vina (version 1.2.3), employing a flexible‐ligand/rigid‐receptor protocol. The binding sites were defined based on the positions of the co‐crystallized ligands in the respective crystal structures. Grid box parameters were carefully defined for each protein to ensure accurate targeting of the active sites. The grid box centers and dimensions were set as follows:

1. Bcl‐2 (2O2F): center (14.732, 22.589, 31.447), size (40 × 40 × 40 Å)
2. ABCG2 (6FFC): center (−8.214, 10.562, 25.873), size (40 × 40 × 40 Å)
3. ABCB1 (6C0V): center (5.948, −3.771, 18.664), size (40 × 40 × 40 Å)
4. IκBα/NF‐κB (1NFI): center (12.375, 8.942, 20.156), size (50 × 50 × 50 Å)
5. HSA (1SAO): center (28.611, 14.228, 9.774), size (40 × 40 × 40 Å)
6. HSA (3GJQ): center (−2.458, 6.331, 21.905), size (40 × 40 × 40 Å)

These parameters were defined based on the geometric center of the corresponding co‐crystallized ligands using BIOVIA Discovery Studio Visualizer, ensuring reproducibility and accurate coverage of the binding pockets.

Docking calculations were performed using an exhaustiveness value of 8, while all other parameters were kept at their default settings. For each ligand, nine binding poses were generated, and the top‐ranked pose based on predicted binding affinity (kcal/mol) was selected for further analysis.

To validate the docking protocol, the co‐crystallized ligands were re‐docked into their respective binding sites under identical conditions. The accuracy of the docking procedure was evaluated by calculating the root‐mean‐square deviation (RMSD) between the docked and experimental poses. The RMSD value obtained for the re‐docked ligand was 1.63 Å, indicating a reliable and robust docking protocol.

Docking results were ranked according to the scoring function implemented in AutoDock Vina, and predicted binding affinity values were used for comparative analysis within the compound series. Protein–ligand interactions were analyzed based on binding orientation and the presence of hydrogen bonds, π–π interactions, and hydrophobic contacts using BIOVIA Discovery Studio Visualizer (version 2024).

## 3 Results and Discussion

### 3.1 Cytotoxic Activity of 3′,4′,5′‐Trimethoxychalcones

Chalcone cytotoxic activity varied markedly among the tested cancer cell lines (Table 1), demonstrating a strong dependence on cellular background. Because all assays were conducted under strictly standardized conditions, the observed differences are more likely to reflect intrinsic cell‐line–dependent responses rather than methodological variability.

**TABLE 1:** Substituent patterns and cytotoxic activity (IC_50_) of chalcone derivatives against human tumor cell lines.

Chalcone	R^1^	R^2^	R^3^	R^4^	R^5^	MDA‐MB‐231^a^	MCF‐7^b^	SKW‐3^c^	K‐562^d^
						
						IC_50_, µM(CI)^e^			
**1**	H	H	H	H	H	46.3 (40.5–52.8)	4.1 (1.3–7.7)	1.9 (1.3–2.7)	4.3 (3.9–4.8)
**2**	H	H	NO_2_	H	H	11.5 (9.8–13.6)	2.7 (1.8–3.5)	1.8 (1.2–2.9)	14.2 (12.2–16.6)
**3**	H	H	CN	H	H	22.4 (18.9–26.4)	14.8 (9.6–22.8)	4.5 (3.5–5.3)	>100
**4**	H	H	CF_3_	H	H	16.9 (14.1–20.2)	0.23 (0.16–0.33)	0.08 (0.07–0.08)	7.7 (4.3–10.8)
**5**	H	H	F	H	H	14.6 (13.3–16.1)	0.29 (0.21–0.49)	0.04 (0.01–0.12)	3.3 (1.8–6.0)
**6**	H	H	Cl	H	H	33.4 (23.3–47.8)	5.6 (4.3–7.3)	10.0 (8.2–12.2)	30.9 (19.6–48.6)
**7**	H	H	Me	H	H	17.6 (15.0–19.8)	7.9 (5.0–9.3)	0.23 (0.20–0.26)	13.0 (10.6–16.1)
**8**	H	H	OMe	H	H	5.4 (4.9–13.2)	0.49 (0.23–1.04)	0.18 (0.17–0.19)	83.0 (47.6–144.8)
**9**	H	H	NMe_2_	H	H	17.3 (14.0–21.4)	0.06 (0.04–0.09)	0.77 (0.68–0.82)	4.1 (2.9–6.0)
**10**	H	H	NEt_2_	H	H	16.2 (13.7–19.1)	0.56 (0.42–0.74)	0.06 (0.04–0.09)	5.3 (3.6–7.7)
**11**	H	H	NHAc	H	H	16.1 (11.7–22.2)	7.6 (5.5–10.4)	3.2 (1.5–7.0)	56.7 (35.6–90.4)
**12**	F	H	H	F	H	17.7 (15.5–20.2)	0.83 (0.51–1.37)	3.4 (2.4–4.6)	12.1 (10.7–15.1)
**13**	F	H	H	CF_3_	H	1.1 (0.8–1.6)	0.12 (0.08–0.27)	0.15 (0.12–0.17)	7.8 (4.5–13.6)
**14**	Cl	H	H	NO_2_	H	3.7 (2.6–5.2)	0.12 (0.09–0.30)	0.16 (0.13–0.20)	14.2 (8.8–22.9)
**15**	OH	H	H	NO_2_	H	8.7 (7.8–9.5)	0.32 (0.19–0.53)	0.08 (0.07–0.09)	2.6 (1.6–5.6)
**16**	OH	H	H	Br	H	2.1 (1.5–2.8)	0.17 (0.14–0.20)	0.08 (0.07–0.09)	2.3 (1.1–3.5)
**17**	H	OMe	H	OMe	H	5.7 (4.8–6.8)	0.80 (0.63–0.99)	0.62 (0.57–0.67)	6.7 (3.8–11.9)
**18**	H	OH	OMe	H	H	36.9 (22.3–61.1)	0.70 (0.29–1.37)	0.18 (0.16–0.19)	25.2 (21.2–30.0)
**19**	H	OMe	OMe	H	H	>100	5.4 (4.1–7.2)	28.5 (19.8–41.0)	>100
**20**	H	─OCH_2_O─		H	H	3.0 (1.7–4.8)	5.6 (3.8–8.1)	0.30 (0.25–0.35)	74.8 (58.4–93.2)
**21**	H	─CH═CH─NH─		H	H	39.9 (35.1–45.3)	1.6 (1.1–2.4)	2.0 (1.3–2.9)	1.4 (1.2–1.6)
**22**	Me	H	Me	H	Me	11.4 (7.6–16.9)	17.6 (12.7–24.3)	2.3 (1.4–3.9)	84.6 (48.3–148.2)
**23**	OMe	H	OMe	H	OMe	73.0 (58.7–88.4)	0.14 (0.04–0.30)	102 (58–180)	43.6 (32.4–58.7)
**24**	H	OMe	OH	OMe	H	18.7 (15.9–22.0)	0.35 (0.33–0.47)	0.02 (0.01–0.13)	6.3 (4.6–8.5)
**25**	H	OMe	OMe	OMe	H	1.2 (0.7–2.0)	7.9 (5.2–10.8)	2.7 (0.5–13.4)	43.9 (27.9–69.2)
Melphalan						27.8 (21.2–32.1)	31.7 (24.4–40.0)	18.2 (12.3–31.4)	21.9 (17.6–33.1)

^a^ Estrogen receptor–negative, ER^–^, breast carcinoma.

^b^ Estrogen receptor–positive, ER^+^, breast carcinoma.

^c^ T‐cell leukemia.

^d^ Erythroleukemia with BCR‐ABL fusion (Philadelphia chromosome–positive).

^e^ IC_50_ values are reported with 95% confidence intervals (CI) given in parentheses. Values denoted as >100 or >200 indicate that IC_50_ could not be determined within the highest concentration tested.

Abbreviations: Me, methyl; OMe, methoxy; NMe_2_, dimethylamino; NEt_2_, diethylamino; NHAc, acetamido; ─OCH_2_O─, methylenedioxy bridge, and ─CH═CH─NH─, aminoethenyl bridge.

All evaluated chalcones exhibited higher cytotoxic potency in ER^+^ MCF‐7 cells than the reference drug melphalan, with several derivatives displaying submicromolar IC_50_ values. Notably, the *p*‐dimethylamino‐substituted chalcone (**9**) showed particularly high cytotoxic activity in this model (IC_50_ = 0.06 µM).

In contrast, the ER^−^ MDA‐MB‐231 displayed a more heterogeneous response profile. Five derivatives were less active than melphalan, whereas the remaining compounds showed comparable or only modestly enhanced cytotoxic potency. Overall sensitivity was markedly lower than that observed in MCF‐7 cells, consistent with differences between ER^+^ and ER^−^ cellular backgrounds. Although ER^+^ MCF‐7 cells exhibited higher overall sensitivity, the present data do not support an estrogen receptor–mediated mechanism, indicating that ER status modulates cellular susceptibility rather than the molecular mode of action. While the present data do not allow attribution of cytotoxicity to a specific molecular target, the observed subtype‐dependent activity is compatible with ER‐independent mechanisms commonly reported for chalcones, such as disruption of microtubule dynamics or induction of cellular stress responses, which are known to be strongly influenced by cellular context [2, 5, 21].

Among the hematological models, SKW‐3 cells showed the highest sensitivity. Most chalcones exceeded the activity of melphalan, with only two derivatives showing lower potency than the reference drug. Several compounds exhibited submicromolar IC_50_ values, including low‐nanomolar activity (0.02–0.08 µM) for derivatives bearing 4‐hydroxy‐3,5‐dimethoxy (**24**), *p*‐fluoro (**5**), *p*‐trifluoromethyl (**4**), 2‐hydroxy‐5‐nitro (**15**), and 2‐hydroxy‐5‐bromo (**16**) modifications on ring B. The presence of structurally diverse active motifs suggests a generally high intrinsic susceptibility of SKW‐3 cells to the chalcone scaffold rather than a narrow preference for specific ring‐B features.

By contrast, K‐562 cells expressing the BCR–ABL oncogenic tyrosine kinase were the least responsive model. Nine derivatives were less active than melphalan, while several others exhibited only moderate cytotoxicity or IC_50_ values exceeding the highest concentration tested. This restricted activity profile suggests that cytotoxic effects in K‐562 cells are observed for a narrower subset of chalcone structures and appear less tolerant to variation on the second aromatic ring.

Taken together, these findings demonstrate that the cytotoxic activity of 3’,4’,5’‐trimethoxychalcones is strongly cell‐line dependent and cannot be described by a single potency ranking across different cancer models. When benchmarked against the clinically used alkylating agent melphalan, the series exhibits pronounced shifts in relative efficacy that primarily reflect differences in cellular susceptibility rather than uniform effects driven solely by the substitution pattern.

A limitation of the present study is the absence of evaluation on noncancerous cell lines, which precludes assessment of selectivity and therapeutic window. Therefore, the observed cytotoxic potency should be interpreted with caution. The inclusion of melphalan as a reference compound provides a comparative benchmark under identical conditions, but does not address selectivity.

### 3.2 Cytotoxic Activity in Drug‐Resistant and Additional Cancer Cell Models

Based on the primary cytotoxicity screening across four cancer cell lines, a subset of the most potent and structurally representative chalcones (**5**, **10**, **15**, **16**, and **24)** was selected for evaluation in HL‐60 and HL‐60/DOX leukemia models to assess activity in a drug‐resistant leukemia context (Table 2). As expected for the resistant phenotype, most compounds showed reduced cytotoxic activity in HL‐60/DOX cells relative to the parental line.

**TABLE 2:** Cytotoxic activity of selected chalcones against sensitive and drug‐resistant human cancer cell lines.

Chalcone	HL60^a^	HL60/DOX^b^	SW‐1710^c^	Cal 29^d^
**5**	5.9 (4.6–7.6)^e^	11.4 (4.5–26.7)	n.d.^f^	n.d.
**10**	0.32 (0.26–0.40)	0.31 (0.26–0.37)	n.d.	n.d.
**15**	7.9 (5.8–10.8)	15.3 (6.8–34.2)	n.d.	n.d.
**16**	5.7 (4.8–6.8)	0.64 (0.40–1.02)	1.5 (1.1–2.2)	> 25
**24**	21.1 (16.0–27.8)	> 25	n.d.	n.d.

^a^ Human promyelocytic leukemia cell line.

^b^ Doxorubicin‐resistant subline, overexpresses the multidrug resistance (MDR)–associated protein MRP‐1.

^c^ Human bladder carcinoma cell line derived from muscle‐invasive urothelial carcinoma.

^d^ Human bladder carcinoma cell line derived from high‐grade urothelial carcinoma.

^e^ IC_50_ values are reported with 95% confidence intervals (CI) given in parentheses.

^f^ n.d.: Not done.

Notably, **10** retained comparable submicromolar activity in both models, whereas **16** was more active in the resistant subline, showing an approximately nine‐fold potency increase relative to HL‐60 cells. However, this differential sensitivity cannot be unambiguously attributed to altered MDR mechanisms and may also reflect differences in proliferation rate, cellular redox state, metabolic capacity, or transporter systems beyond MRP‐1. To further clarify the underlying mechanisms, follow‐up studies such as efflux inhibition assays and measurements of ROS generation and mitochondrial function would be informative. These observations indicate that cytotoxic responses within this selected subset are not uniformly attenuated in the DOX‐adapted model and are compatible with modes of action that are not strictly governed by classical MDR mechanisms.

Retention or partial preservation of cytotoxic activity in leukemia cells with altered drug‐response profiles has previously been reported for selected chalcone derivatives, particularly in studies addressing interference with drug‐efflux mechanisms and modulation of cellular response [1]. More broadly, reviews of chalcone cytotoxicity and other α,β‐unsaturated carbonyl compounds describe instances in which classical resistance mechanisms do not uniformly abolish activity, underscoring the pronounced cell‐line dependence of cytotoxic outcomes across different cancer models [4, 31, 32].

In addition, chalcone **16** was evaluated against two human bladder carcinoma cell lines. The compound displayed micromolar cytotoxicity toward SW‐1710 cells, whereas no significant activity was detected in Cal‐29 cells within the tested concentration range (Table 2). Such divergent responses in closely related solid tumor models are consistent with earlier reports on chalcone derivatives and other small‐molecule cytotoxins and further underscore the dominant influence of tissue‐ and lineage‐specific cellular context on cytotoxic efficacy [1, 2].

### 3.3 Structure–Activity Relationships and Context Dependence

#### 3.3.1 Hierarchical Clustering and PCA Analysis

**FIGURE 1:**
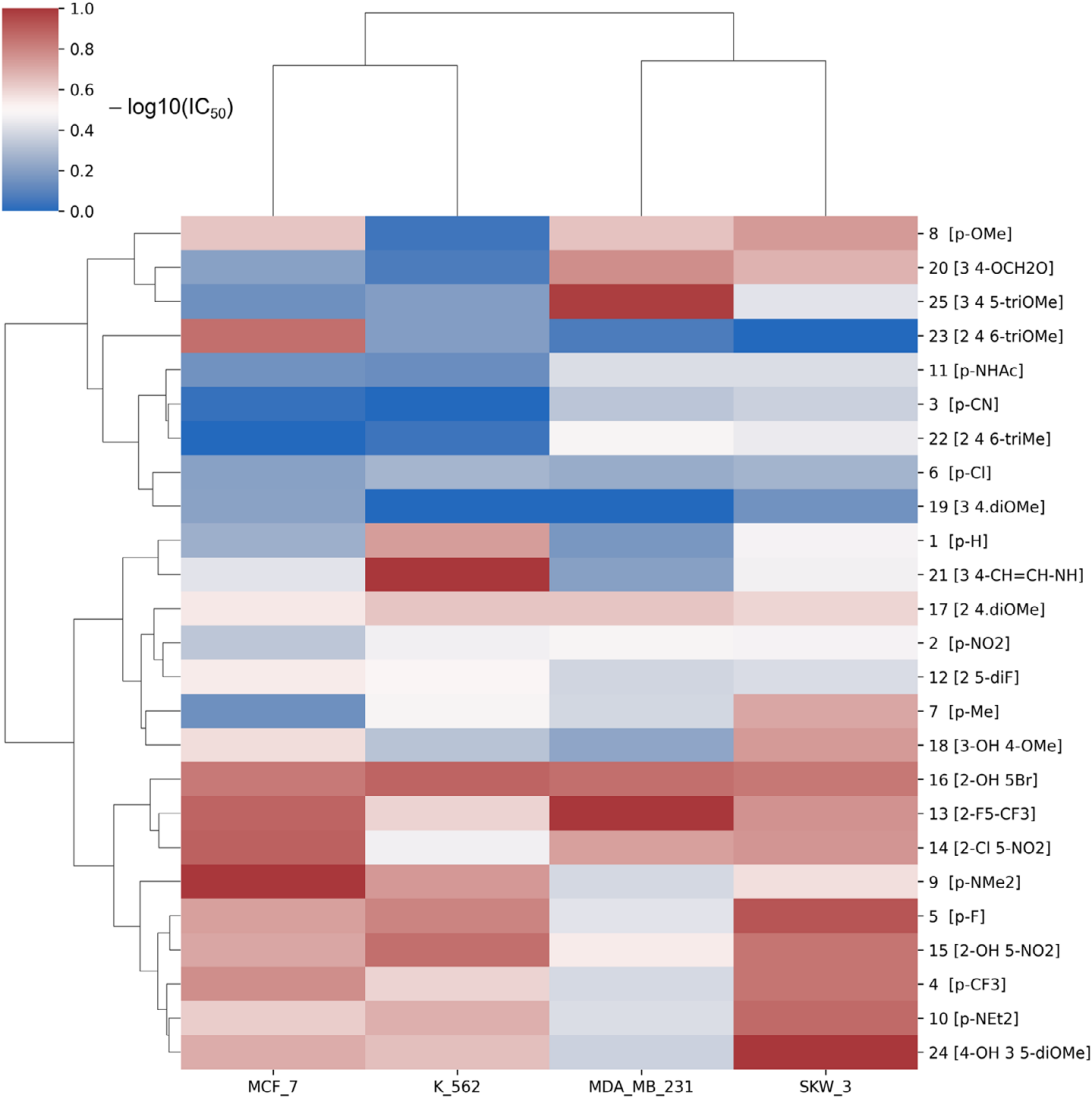
Hierarchical clustering of cytotoxic activity profiles of chalcone derivatives across cancer cell lines. The heatmap was generated using −log_10_‐transformed IC_50_ values, z‐score normalized within each cell line to emphasize relative activity patterns. Red denotes higher‐than‐average cytotoxic activity (lower IC_50_), whereas blue indicates lower activity. Unsupervised hierarchical clustering was performed for both compounds and cell lines using Ward's method with Euclidean distance.

To explore structure–activity relationships (SARs) within the investigated 3′,4′,5′‐trimethoxychalcone series and to assess their dependence on cellular context, hierarchical clustering of the cytotoxicity data was performed (Figure 1). The resulting heatmap revealed distinct clustering of both chalcone derivatives and cancer cell lines, reflecting pronounced differences in relative cytotoxic response profiles and underscoring the importance of biological context in shaping activity patterns.

Unsupervised clustering of −log_10_‐transformed IC_50_ profiles grouped compounds according to similarity in cytotoxic behavior across the cancer cell lines included in Table 1, separating derivatives with relatively consistent activity patterns from those exhibiting weak or pronounced cell‐line selectivity. The most active derivatives form a coherent subgroup characterized by uniformly elevated −log_10_(IC_50_) values across the tested models, whereas other clusters display heterogeneous and context‐dependent response patterns, highlighting functional diversity within the compound library.

**FIGURE 2:**
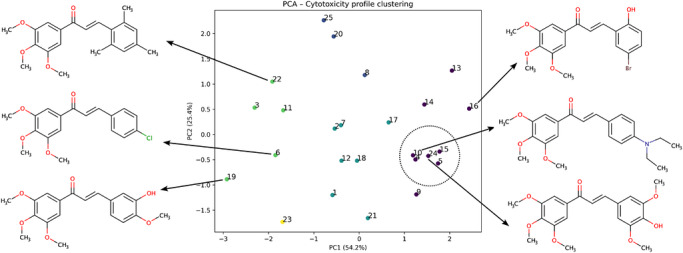
Principal component analysis (PCA) of ‐log_10_(IC_50_) cytotoxicity data for chalcone derivatives. Points represent individual compounds, colored by unsupervised cluster membership. Representative molecular structures are shown for chemically and biologically distinct regions of the PCA space. The circled region indicates a compact activity cluster with similar cytotoxic profiles.

Complementary to hierarchical clustering, PCA was applied to the same −log_10_(IC_50_) dataset to provide a low‐dimensional representation of cytotoxicity profiles (Figure 2). The first two principal components (PC1 and PC2) together accounted for approximately 80% of the total variance in the dataset. The PCA projection reveals a structured distribution of compounds according to similarity in cytotoxic profiles, with derivatives sharing comparable response patterns clustering in close proximity in principal component space. Variation along PC1 is dominated by differences in overall cytotoxic response magnitude, whereas PC2 reflects differences in relative sensitivity among the tested cell lines, emphasizing context‐dependent activity trends. Compounds exhibiting relatively consistent cytotoxic responses across multiple cell lines localize within a compact region of the score plot, while less active or more selective derivatives are distributed across distinct regions, reflecting divergent activity signatures. Although representative chalcone structures are shown in the PCA plot for qualitative orientation, the analysis is based exclusively on cytotoxicity data and reflects similarities in biological response profiles rather than mechanistic structure–activity relationships.

#### 3.3.2 Substitution Patterns on Ring B

Across the examined substitution motifs on ring B—including *p*‐monosubstituted, 2,5‐ and 3,4‐disubstituted, and trisubstituted derivatives—cytotoxic potency was strongly dependent on cellular context rather than on a single substituent class. Accordingly, no universal substituent‐driven SAR emerged across the full panel of tested cell lines, consistent with the weak correlations (R^2^ ≤ 0.02) observed for global molecular descriptors such as dipole moment (Section 3.3.3).

Given the absence of a general SAR, further analysis was restricted to defined substitution subsets and the most sensitive cell lines. In MCF‐7 cells, the *p*‐monosubstituted ring‐B series (**1**–**11**) exhibits a clear potency order: *p*‐NMe_2_ (**9**) ≫ *p*‐CF_3_ (**4**) > *p*‐F (**5**) > *p*‐OMe (**8**) ≈ *p*‐NEt_2_ (**10**) ≫ *p*‐NO_2_ (**2**) > H (**1**) > *p*‐Cl (**6**) ≈ *p*‐NHAc (**11**) ≈ *p*‐Me (**7**) ≫ *p*‐CN (**3**). Activity is maximized by compact basic or low‐polarity lipophilic *p‐*substituents, whereas bulky amines and highly polar or linear groups are disfavored; electron‐withdrawing strength alone does not predict potency. This behavior is consistent with reported vesicular acidification defects in MCF‐7 cells that enhance sensitivity to substituent‐driven changes in effective intracellular exposure [33].

Additional substitution on ring B modulates MCF‐7 activity in a pattern‐dependent manner. For *p*‐OMe‐based derivatives, potency varies with substitution geometry: 2,4,6‐triOMe (**23**) > 3‐OH;4‐OMe (**18**) > *p*‐OMe (**8**) > 3,4‐diOMe (**19**) > 3,4,5‐triOMe (**25**). Accordingly, replacement of the *p*‐OMe group in compound **25** with a *p*‐OH substituent enhanced the cytotoxic activity of the resulting analogue **24** by approximately 23‐fold. Although increased methoxylation has frequently been associated with enhanced chalcone cytotoxicity, the present results demonstrate that this effect is not universal and depends critically on the number and positional arrangement of methoxy groups, particularly in MCF‐7 cells [1, 5]. Within the 2,5‐disubstituted series, activity decreases as F/CF_3_ (**13**) > OH/Br (**16**) > Cl/NO_2_ (**14**) > OH/NO_2_ (**15**) > F/F (**12**), indicating a preference for mixed hydrophobic/polarizable substitution patterns, while symmetric halogen motifs are less favorable.

In SKW‐3 cells, the *p*‐monosubstituted ring‐B series (**1**–**11**) follows a different potency order: *p*‐F (**5**) > *p*‐NEt_2_ (**10**) > *p*‐CF_3_ (**4**) > *p*‐OMe (**8**) > *p*‐Me (**7**) > *p*‐NMe_2_ (**9**) > *p*‐NO_2_ (**2**) > H (**1**) > *p*‐NHAc (**11**) > *p*‐CN (**3**) > *p*‐Cl (**6**). Here, activity is maximized by compact, low‐polarity hydrophobic substituents, with halogenated derivatives occupying the top tier. Dialkylamino groups are tolerated but display a size dependence opposite to that observed in MCF‐7, as p‐NEt_2_ is markedly more potent than p‐NMe_2_, suggesting a benefit of increased hydrophobic bulk in this cell line. In contrast, polar or strongly solvated substituents are disfavored.

Beyond *p‐*monosubstitution, SKW‐3 cells also show pronounced pattern‐dependent sensitivity to additional substitution on ring B. Within the 2,5‐disubstituted series, activity follows the order: OH/Br (**16**) ≈ OH/NO_2_ (**15**) > F/CF_3_ (**13**) ≈ Cl/NO_2_ (**14**) ≫ F/F (**12**), indicating that asymmetric substitution patterns combining hydrophobic and polarizable or hydrogen‐bonding groups are favored, whereas symmetrical halogenation is strongly disfavored. Similarly, in the 3,4‐disubstituted series, potency decreases as 3‐OH/4‐OMe (**18**) > 3,4‐(OCH_2_O) (**20**) ≫ 3,4‐diOMe (**19**), underscoring the importance of substituent complementarity and polarity distribution rather than substituent identity alone.

From a comparative perspective, certain substituent effects are conserved across models within defined subsets (e.g., *p*‐monosubstitution) in the two most sensitive cell lines; however, the overall potency ranking remains strongly cell‐line dependent, in agreement with previous reports on the context‐dependent behavior of 3,4,5‐chalcone‐based anticancer agents [1, 5, 8, 31, 32].

#### 3.3.3 Descriptor‐Based QSAR

No meaningful correlations (R^2^ ≤ 0.14) were observed between cytotoxic IC_50_ values and global physicochemical or structural parameters, including molecular size, lipophilicity, and surface‐related descriptors. This indicates that cytotoxic effects within the present chalcone series are not adequately explained by simple bulk molecular properties and strongly influenced by cell‐line‐specific biological context.

Previous QSAR investigations of chalcones have shown that statistically significant correlations between cytotoxic activity and physicochemical or electronic descriptors may emerge within restricted compound sets or when analyses are confined to individual cell lines. For example, Sakagami et al. identified quantitative relationships involving electronic and steric parameters in specific cellular models, while also demonstrating that these correlations deteriorate when extended to structurally diverse compounds or multiple cell systems [12].

In line with these observations, more recent computational studies employing machine‐learning and chemical space analyses emphasize the multiplicity of interaction patterns underlying chalcone cytotoxicity, rather than a single dominant molecular determinant, thereby highlighting the limited predictive performance of traditional descriptor‐based models in chemically diverse datasets [34].

### 3.4 Molecular Docking Studies

#### 3.4.1 Docking to Cancer‐Relevant Protein Targets

In light of the limited explanatory power of descriptor‐based QSAR models and the pronounced cell‐line‐dependent variability in cytotoxic responses, molecular docking was applied as a complementary, structure‐based approach to probe plausible interaction modes of selected highly active chalcones (**5**, **10**, **15**, **16**, and **24**) with cancer‐relevant protein targets. The chosen targets are involved in biological processes previously implicated in chalcone activity, including regulation of mitochondrial apoptosis (Bcl‐2), transcriptional control of cell survival and inflammation (IκBα/NF‐κB complex), and ATP‐dependent drug efflux associated with multidrug resistance (ABCB1/P‐gp and ABCG2/BCRP) (Table 3) [6, 7, 15, 35].

**TABLE 3:** Molecular docking results of selected highly active chalcones against cancer‐relevant protein targets. Selected chalcones (5, 10, 15, 16, and 24) were chosen based on their high cytotoxic potency in one or more cancer cell lines (Table 1). Docking was performed against protein targets implicated in apoptosis regulation, transcriptional control, and multidrug resistance. Binding energies are reported in kcal/mol.

Target (PDB)	Protein target	Biological role/ relevance	5	10	15	16	24
2O2F	Bcl‐2	Anti‐apoptotic regulator of mitochondrial cell death	−5.9	−5.7	−6.2	−5.8	−6.1
6FFC	ABCG2 (BCRP)	ATP‐dependent drug efflux transporter involved in multidrug resistance	−7.3	−7.1	−7.4	−7.5	−6.9
6C0V	ABCB1 (P‐gp)	ATP‐dependent drug efflux transporter involved in multidrug resistance	−7.7	−7.2	−8.2	−8.1	−7.8
1NFI	IκBα / NF‐κB complex	Transcriptional regulation of cell survival, inflammation, and immune responses	−6.5	−6.3	−6.6	−6.6	−6.4

**FIGURE 3:**
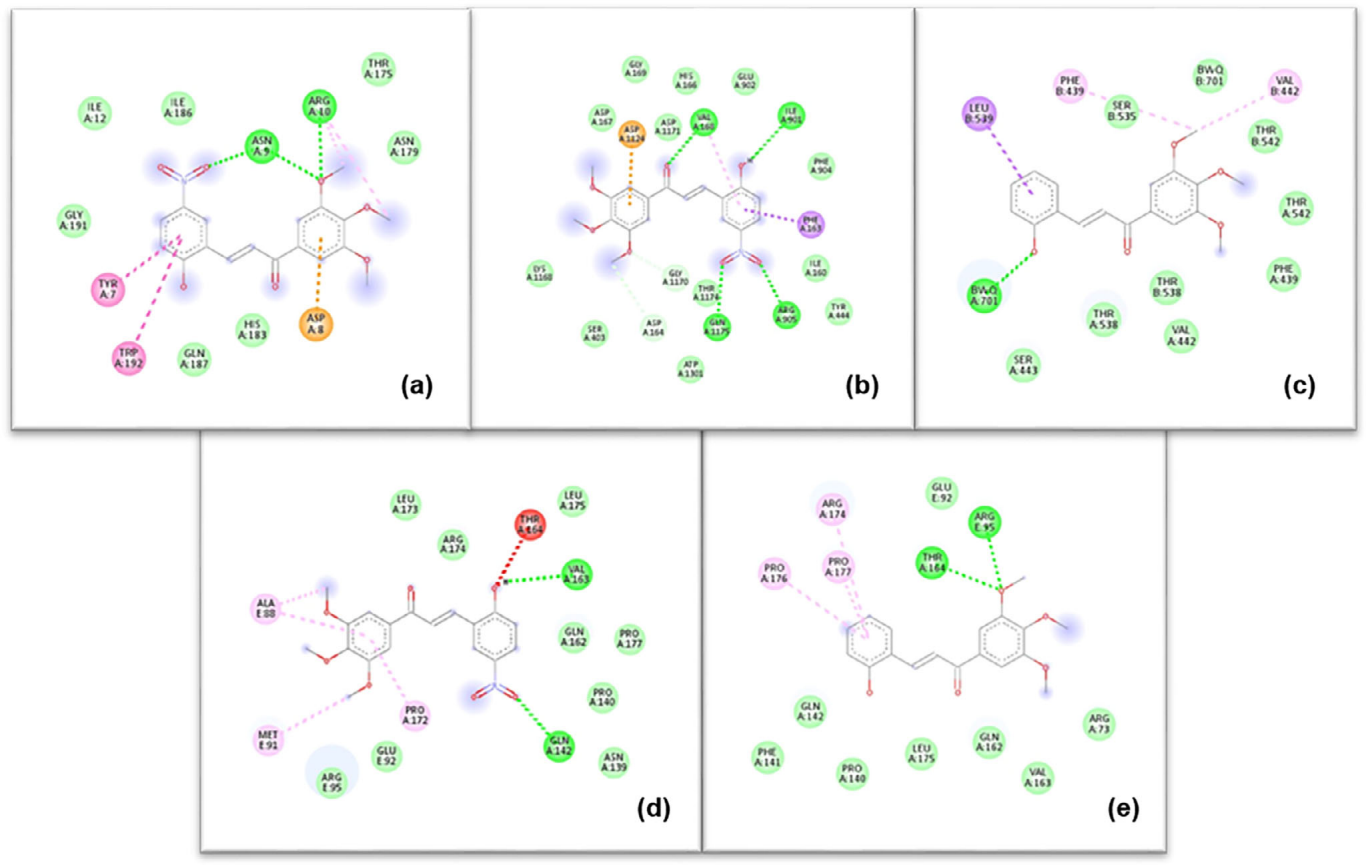
Two‐dimensional docking models illustrating the interactions formed by chalcone 15 and chalcone 16 with selected protein targets. (a) Chalcone 15 interacts with Bcl‐2 via π–anion interaction with ASP A:8 and hydrogen bonding with ASN A:9 and ARG A:10. (b) Chalcone 15 interacts with P‐gp (ABCB1) through π–σ interaction with PHE A:163, π–anion interaction with ASP A:1124, and hydrogen bonding with VAL A:168, ILE A:901, and ARG A:905. (c) Chalcone 16 interacts with ABCG2 via a π–σ interaction with LEU B:539. (d) Chalcone 15 interacts with the IκBα/NF‐κB complex through hydrogen bonding with GLN A:142 and VAL A:163. (e) Chalcone 16 interacts with the IκBα/NF‐κB complex via hydrogen bonding with ARG E:95 and THR A:164.

Chalcones **15** and **16** were selected for more detailed visualization due to their consistently low docking energies and interaction‐rich binding modes across several targets (Figure 3).

The interaction maps indicate that the investigated chalcones engage the selected protein targets predominantly through hydrophobic, π–π, and π–alkyl interactions, with occasional hydrogen‐bond contributions depending on the local binding environment. No individual target exhibited a distinctive interaction pattern or binding advantage that could account for the observed differences in cytotoxic potency among the compounds. Instead, the docking results suggest that the 3′,4′,5′‐trimethoxychalcone scaffold is broadly compatible with moderate, chemically plausible interactions across multiple cancer‐relevant proteins.

Although the selected chalcones exhibited moderate and chemically plausible binding to multiple cancer‐relevant targets, these interactions did not differentiate highly active from weakly active compounds and therefore do not account for the observed cell‐line‐dependent cytotoxicity. Instead, differential cytotoxic activity within this series reflects context‐dependent cellular factors rather than preferential engagement of a single molecular target.

#### 3.4.2 Human Serum Albumin (HSA) Docking

**FIGURE 4:**
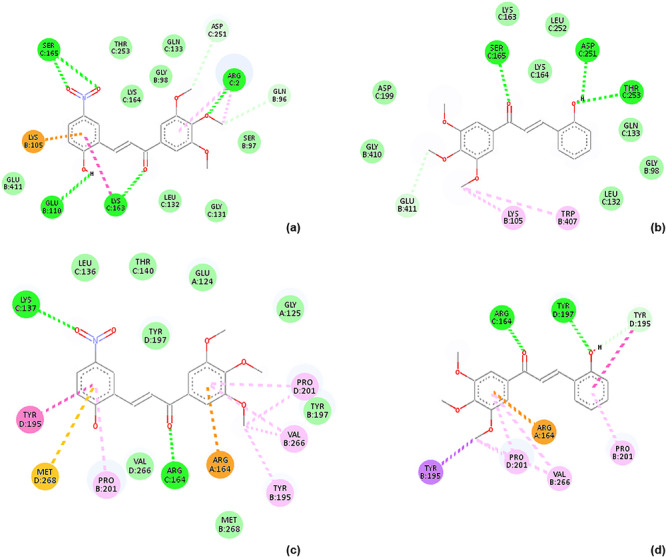
Two‐dimensional docking models illustrating the interactions formed by chalcones 15 and 16 with human serum albumin (HSA) using two crystallographic structures (PDB IDs: 1SAO and 3GJQ). (a) Chalcone 15 interacts with HSA (1SAO) through a π–cation interaction with LYS B:105, amide–π stacking with LYS C:163, alkyl and π–alkyl interactions with ARG C:2, hydrogen bonding with SER C:165, GLU B:110, LYS C:163, and ARG C:2, and van der Waals interactions with ASP C:251 and GLN B:96. (b) Chalcone **16** interacts with HSA (1SAO) through hydrogen bonding with SER C:165, ASP C:251, and THR C:253, alkyl interactions with LYS B:105, π–alkyl interactions with TRP B:407, and van der Waals interactions with GLU B:411. (c) Chalcone 15 interacts with HSA (3GJQ) through π–cation interaction with ARG A:164, π–sulfur interaction with MET D:268, π–π stacking with TYR D:195, alkyl interactions with PRO D:201 and VAL B:266, π–alkyl interactions with PRO B:201, PRO D:201, and VAL B:266, and hydrogen bonding with LYS C:137 and ARG A:164. (d) Chalcone 16 interacting with HSA (3GJQ) via a π–σ interaction with TYR B:195, π–cation interaction with ARG A:164, alkyl interactions with PRO D:201 and VAL B:266, π–alkyl interactions with PRO D:201, PRO B:201, and VAL B:266, hydrogen bonding with ARG C:164 and TYR D:197, and van der Waals interactions with TYR D:195.

Docking to human serum albumin (HSA) was performed to assess whether differences in cytotoxic activity within the chalcone series could plausibly be associated with differential albumin binding and, by extension, with variations in transport or distribution, given the central role of HSA in drug binding and systemic bioavailability [36, 37]. Representative two‐dimensional docking poses of chalcones **15** and **16** are shown in Figure 4, illustrating comparable binding modes within two distinct ligand‐bound conformations of the protein.

Consistent with these observations, all investigated chalcones exhibited similar binding affinities toward HSA (Table 4). These results indicate that albumin interaction represents a shared, scaffold‐related property of the 3′,4′,5′‐trimethoxychalcone series and does not differentiate compounds with respect to cytotoxic potency. Within the scope of this analysis, HSA docking therefore serves to delimit the contribution of serum protein binding as a discriminating factor underlying the observed activity differences among the investigated derivatives.

**TABLE 4:** Molecular docking results of selected highly active chalcones (5, 10, 15, 16, and 24) against cancer‐relevant protein targets. Binding energies are given in kcal/mol.

Target (PDB)	Protein target	Biological role/ relevance	5	10	15	16	24
1SAO^a^	Human serum albumin (HSA)	Plasma transport protein; drug binding and distribution	−7.0	−7.2	−7.1	−7.6	−7.2
3GJQ	Human serum albumin (HSA)	Plasma transport protein; drug binding and distribution	−7.3	−7.2	−7.2	−7.8	−6.7

^a^ PDB structures 1SAO and 3GJQ represent different ligand‐bound conformations of human serum albumin and were used to account for protein flexibility in ligand binding.

Although trimethoxy‐substituted chalcones have been reported to interact with the colchicine‐binding site of tubulin [5, 6, 7, 8], the present docking analyses were not intended to exhaustively survey all proposed chalcone targets. Instead, they were used to examine whether differential binding to selected cancer‐relevant proteins or to serum albumin could plausibly account for the observed variability in cytotoxic activity. Within these limits, no dominant target interaction or differential albumin binding emerged, supporting a multifactorial and context‐dependent basis for the observed activity patterns.

## 4 Conclusion

This study identified multiple potent 3′,4′,5′‐trimethoxychalcone derivatives. Their cytotoxic activity is governed primarily by cellular context rather than by universal, substitution‐driven SAR rules. Under identical experimental conditions, most investigated chalcones exceeded the cytotoxic potency of melphalan. Several derivatives showed pronounced activity in the most responsive cancer models. Distinct local SAR trends were evident in MCF‐7 cells, whereas SKW‐3 cells displayed largely scaffold‐driven sensitivity with limited dependence on specific substitution patterns. In contrast, MDA‐MB‐231 and K‐562 cells required particular or reinforced motifs to achieve measurable cytotoxic responses. Accordingly, ring‐B modification primarily modulates biological response profiles rather than uniformly enhancing cytotoxic potency. Hierarchical clustering, PCA, descriptor‐based QSAR, and molecular docking indicate that cytotoxic activity within this chalcone series reflects a multifactorial, context‐dependent mode of action that cannot be explained by simple physicochemical descriptors or single‐target interactions.

## Author Contributions

**Aleksandar Mehandzhiyski**: methodology, conceptualization, software, writing – review and editing. **Abdessamad Beraich**: methodology, visualization, software. **Zhivko Velkov**: methodology, conceptualization, writing – review and editing. **Maya Zaharieva**: methodology, writing – review and editing. **Spiro Konstantinov**: methodology, writing – review and editing. **Daniela Batovska**: supervision, conceptualization, methodology, writing – original draft, review and editing.

## Funding

The authors have nothing to report.

## Conflicts of Interest

The authors declare no conflicts of interest.

## Supporting information




**Supporting File**: cbdv71336‐sup‐0001‐SuppMat.docx

## Data Availability

The data that support the findings of this study are available from the corresponding author upon reasonable request.
