# Supplementary material for: Cytotoxic Profiling of 3′,4′,5′‐Trimethoxychalcones Reveals Cell‐Line‐Dependent Cytotoxic Activity: An In Vitro and In Silico Study
Source: Chem Biodivers. 2026 May 13;23:e71336. doi: 10.1002/cbdv.71336 (PMC13172435; doi:10.1002/cbdv.71336)
Supplement: Supplementary file 1 — Supporting File: cbdv71336‐sup‐0001‐SuppMat.docx [file CBDV-23-e71336-s001.docx]

**Supplementary Information**

**Cytotoxic Profiling of 3’,4’,5’-Trimethoxychalcones Reveals Cell-Line-Dependent Cytotoxic Activity: An In Vitro and In Silico Study**

Aleksandar Y. Mehandzhiyski *^[a]^, Abdessamad Beraich ^[b]^, Zhivko Velkov ^[c]^, Maya Zaharieva ^[d]^, Spiro Konstantinov ^[e]^, Daniela Batovska *^[f]^

[a] Dr., AM, Mehandzhiyski
Laboratory of Organic Electronics, Department of Science and Technology (ITN)

Linköping University
Campus Norrköping, 601 74 Norrköping, Sweden

email: aleksandar.mehandzhiyski@liu.se

[b] PhD Student, AB, Beraich
Team: Physical Chemistry of the Natural Resources and Processes, Department of Chemistry, Faculty of Sciences
Mohamed First University
Oujda 60000, Morocco

[c] Assoc. Prof. Dr., ZV, Velkov
Department of Chemistry

South-West University “Neofit Rilski”,

66 Ivan Mihajlov, 2700, Blagoevgrad, Bulgaria

[d] Assoc. Prof. Dr., MZ, Zaharieva
Department of Infectious Microbiology, The Stephan Angeloff Institute of Microbiology
Bulgarian Academy of Sciences
Acad. G. Bonchev Str. Bl. 26, 1113 Sofia, Bulgaria

[e] Prof. DSc, SK, Konstantiinov
Department of Pharmacology, Pharmacotherapy and Toxicology
Medical University of Sofia, Faculty of Pharmacy
2 Dunav Str., 1000 Sofia, Bulgaria

[f] Prof. Dr., DB, Batovska
Institute of Chemical Engineering
Bulgarian Academy of Sciences
Acad. G. Bonchev Str., Bl. 103, 1113 Sofia, Bulgaria
E-mail: danibat@iche.bas.bg

**^1^H and ^13^C NMR data**

(2*E*)-1-(3,4,5-trimethoxyphenyl)-3-phenylprop-2-en-1-one (**1**).

¹H NMR (600 MHz, CDCl₃, 25°C, TMS, ppm): **δ**=7.78 (d, *J*=15.6 Hz, 1H; Hβ), 7.63–7.60 (m, 2H; Ar–H), 7.52–7.48 (m, 2H; Ar–H), 7.42–7.38 (m, 1H; Ar–H), 7.36 (d, *J*=15.6 Hz, 1H; Hα), 6.72 (s, 2H; H-2,6), 3.88 (s, 6H; OCH₃-3,5), 3.82 (s, 3H; OCH₃-4); ¹³C NMR (600 MHz, CDCl₃, 25°C, TMS, ppm): **δ**=189.5 (C=O), 153.6 (C-3,5), 146.8 (C-4), 144.1 (Cα), 136.5 (Cβ), 134.8 (Cipso), 130.2, 128.9, 128.5, 127.5 (Ar–CH), 105.8 (C-2,6), 60.9 (OCH₃-4), 56.2 (OCH₃-3,5).

(2*E*)-1-(3,4,5-trimethoxyphenyl)-3-(4-nitrophenyl)prop-2-en-1-one (**2**).

¹H NMR (250 MHz, DMSO-d₆, 25°C, TMS, ppm): **δ**=8.28–8.23 (m, 2H; Ar–H), 7.77 (d, *J*=15.8 Hz, 1H; Hβ), 7.63 (d, *J*=8.5 Hz, 2H; Ar–H), 7.52 (d, *J*=15.8 Hz, 1H; Hα), 7.18 (s, 2H; H-2′,6′), 3.93 (s, 3H; OCH₃-4′), 3.91 (s, 6H; OCH₃-3′,5′); ¹³C NMR (250 MHz, DMSO-d₆, 25°C, TMS, ppm): **δ**=189.3 (C=O), 154.3 (C-3′,5′), 148.1 (C-4), 145.6 (Cβ), 145.2 (C-4′), 139.8 (C-1), 133.1 (C-1′), 128.2 (C-2,6), 122.4 (C-3,5), 121.0 (Cα), 107.8 (C-2′,6′), 56.4 (OCH₃-4′), 56.0 (OCH₃-3′,5′).

(2*E*)-1-(3,4,5-trimethoxyphenyl)-3-(4-cyanophenyl)prop-2-en-1-one (**3**).

¹H NMR (250 MHz, DMSO-d₆, 25°C, TMS, ppm): **δ**=7.77 (d, *J*=15.5 Hz, 1H; Hβ), 7.72–7.68 (m, 2H; Ar–H), 7.60–7.56 (m, 2H; Ar–H), 7.54 (d, *J*=15.5 Hz, 1H; Hα), 7.27 (s, 2H; H-2′,6′), 3.95 (s, 6H; OCH₃-3′,5′), 3.94 (s, 3H; OCH₃-4′); ¹³C NMR (250 MHz, DMSO-d₆, 25°C, TMS, ppm): **δ**=188.4 (C=O), 153.2 (C-3′,5′), 143.0 (C-1), 141.9 (Cβ), 139.2 (C-4′), 132.8 (C-1′), 132.7 (C-3,5), 129.6 (C-2,6), 124.8 (Cα), 118.3 (C≡N), 113.5 (C-4), 106.3 (C-2′,6′), 61.0 (OCH₃-4′), 56.4 (OCH₃-3′,5′).

(2*E*)-1-(3,4,5-trimethoxyphenyl)-3-(4-(trifluoromethyl)phenyl)prop-2-en-1-one (**4**).

¹H NMR (600 MHz, CDCl₃, 25°C, TMS, ppm): **δ**=7.75 (d, *J*=15.6 Hz, 1H; Hβ), 7.68 (d, *J*=8.4 Hz, 2H; Ar–H), 7.62 (d, *J*=8.4 Hz, 2H; Ar–H), 7.48 (d, *J*=15.6 Hz, 1H; Hα), 7.21 (s, 2H; H-2′,6′), 3.89 (s, 6H; OCH₃-3′,5′), 3.88 (s, 3H; OCH₃-4′); ¹³C NMR (600 MHz, CDCl₃, 25°C, TMS, ppm): **δ**=188.8 (C=O), 153.3 (C-3′,5′), 142.7 (Cα), 138.3 (Cβ), 133.1 (C-1), 128.5 (Ar–CH), 126.0 (Ar-CH), 125.9 (Ar–CH), 124.0 (Ar-CH), 106.2 (C-2′,6′), 61.1 (OCH₃-4′), 56.5 (OCH₃-3′,5′).

(2*E*)-1-(3,4,5-trimethoxyphenyl)-3-(4-fluorophenyl)prop-2-en-1-one (**5**).

¹H NMR (600 MHz, CDCl₃, 25°C, TMS, ppm): **δ**=7.72 (d, *J*=15.6 Hz, 1H; Hβ), 7.58–7.54 (m, 2H; Ar–H), 7.34 (d, *J*=15.6 Hz, 1H; Hα), 7.20 (s, 2H; H-2′,6′), 7.05 (t, *J*=8.4 Hz, 2H; Ar–H), 3.89 (s, 6H; OCH₃-3′,5′), 3.88 (s, 3H; OCH₃-4′); ¹³C NMR (600 MHz, CDCl₃, 25°C, TMS, ppm): **δ**=189.0 (C=O), 164.9 (d, J≈250 Hz; C–F), 153.2 (C-3′,5′), 143.5 (Cα), 142.5 (Cβ), 133.4 (C-1), 131.2 (d, J≈8 Hz; Ar–CH), 130.4 (Ar–CH), 121.4 (Ar–CH), 116.2 (Ar–CH), 106.1 (C-2′,6′), 61.0 (OCH₃-4′), 56.4 (OCH₃-3′,5′).

(2*E*)-1-(3,4,5-trimethoxyphenyl)-3-(4-chlorophenyl)prop-2-en-1-one (**6**).

¹H NMR (600 MHz, CDCl₃, 25°C, TMS, ppm): **δ**=7.78 (d, *J*=15.6 Hz, 1H; Hβ), 7.65 (d, *J*=8.6 Hz, 2H; Ar–H), 7.55 (d, *J*=15.6 Hz, 1H; Hα), 7.38 (d, *J*=8.6 Hz, 2H; Ar–H), 6.72 (s, 2H; H-2′,6′), 3.88 (s, 6H; OCH₃-3′,5′), 3.82 (s, 3H; OCH₃-4′); ¹³C NMR (600 MHz, CDCl₃, 25°C, TMS, ppm): **δ**=189.4 (C=O), 153.5 (C-3′,5′), 146.7 (C-4), 143.5 (Cα), 136.8 (Cβ), 135.0 (Cipso–Cl), 132.5, 129.3, 128.8 (Ar–CH), 105.8 (C-2′,6′), 60.9 (OCH₃-4′), 56.2 (OCH₃-3′,5′).

(2*E*)-1-(3,4,5-trimethoxyphenyl)-3-(4-methylphenyl)prop-2-en-1-one (**7**).

¹H NMR (250 MHz, CDCl₃, 25°C, TMS): **δ**=7.79 (d, *J*=15.5 Hz, 1H; Hβ), 7.54 (d, *J*=8.0 Hz, 2H; Ar–H), 7.43 (d, *J*=15.5 Hz, 1H; Hα), 7.22 (d, *J*=8.3 Hz, 2H; Ar–H), 7.27 (s, 2H; H-2′,6′), 3.94 (s, 6H; OCH₃-3′,5′), 3.93 (s, 3H; OCH₃-4′), 2.39 (s, 3H; Ar–CH₃); ¹³C NMR (250 MHz, CDCl₃, 25°C, TMS): **δ**=189.3 (C=O), 153.1 (C-3′,5′), 144.8 (Cα), 142.4 (C-4″), 141.1 (Cβ), 133.6 (C-1), 132.1 (C-1″), 129.7 (C-3″,5″), 128.4 (C-2″,6″), 120.8 (Cα-aryl), 106.1 (C-2′,6′), 60.9 (OCH₃-4′), 56.4 (OCH₃-3′,5′), 21.5 (Ar–CH₃).

(2*E*)-1-(3,4,5-trimethoxyphenyl)-3-(4-methoxyphenyl)prop-2-en-1-one (**8**).

¹H NMR (600 MHz, CDCl₃, 25°C, TMS, ppm): **δ**=7.75 (d, *J*=15.6 Hz, 1H; Hβ), 7.58 (d, *J*=8.6 Hz, 2H; Ar–H), 7.45 (d, *J*=15.6 Hz, 1H; Hα), 6.90 (d, *J*=8.6 Hz, 2H; Ar–H), 6.72 (s, 2H; H-2′,6′), 3.88 (s, 6H; OCH₃-3′,5′), 3.83 (s, 3H; OCH₃-4′), 3.80 (s, 3H; Ar–OCH₃); ¹³C NMR (600 MHz, CDCl₃, 25°C, TMS, ppm): **δ**=189.3 (C=O), 163.0 (C-4″, Ar–OCH₃), 153.5 (C-3′,5′), 146.8 (C-4′), 144.0 (Cα), 136.5 (Cβ), 131.5, 129.0, 128.5 (Ar–CH), 114.5 (Ar–CH), 105.8 (C-2′,6′), 60.9 (OCH₃-4′), 56.2 (OCH₃-3′,5′), 55.3 (Ar–OCH₃).

(2*E*)-1-(3,4,5-trimethoxyphenyl)-3-(4-(dimethylamino)phenyl)prop-2-en-1-one (**9**).

¹H NMR (600 MHz, CDCl₃, 25°C, TMS, ppm): **δ**=7.97–7.96 (m, 2H; Ar–H), 7.64 (d, *J*=15.5 Hz, 1H; Hβ), 7.35 (d, *J*=15.5 Hz, 1H; Hα), 6.92 (m, 2H; Ar–H), 6.79 (s, 2H; H-2′,6′), 3.85 (s, 6H; OCH₃-3′,5′), 3.83 (s, 3H; OCH₃-4′), 3.82 (s, 6H; N(CH₃)₂); ¹³C NMR (600 MHz, CDCl₃, 25°C, TMS, ppm): **δ**=189.7 (C=O), 163.4 (C-4″), 153.5 (C-3′,5′), 144.2 (Cα), 140.2 (Cβ), 131.1, 130.8, 130.6 (Ar–CH), 121.2 (Ar–CH), 113.9 (Ar–CH), 105.5 (C-2′,6′), 61.0 (OCH₃-4′), 56.2 (OCH₃-3′,5′), 55.5 (N(CH₃)₂).

(2*E*)-1-(3,4,5-trimethoxyphenyl)-3-(4-(diethylamino)phenyl)prop-2-en-1-one (**10**).

¹H NMR (600 MHz, CDCl₃, 25°C, TMS, ppm): **δ**=7.80 (d, *J*=15.6 Hz, 1H; Hβ), 7.58–7.55 (m, 2H; Ar–H), 7.28 (d, *J*=15.0 Hz, 1H; Hα), 6.71–6.68 (m, 2H; Ar–H), 6.72 (s, 2H; H-2′,6′), 3.97 (s, 6H; OCH₃-3′,5′), 3.95 (s, 3H; OCH₃-4′), 3.45 (q, *J*=7.2 Hz, 4H; NCH₂), 1.23 (t, *J*=7.2 Hz, 6H; CH₃); ¹³C NMR (600 MHz, CDCl₃, 25°C, TMS, ppm): **δ**=189.5 (C=O), 153.0 (C-3′,5′), 149.7 (C-4″), 145.9 (Cα), 141.8 (Cβ), 134.6 (C-1), 130.8 (Ar–CH), 121.8 (Ar–CH), 115.9 (Ar–CH), 111.3 (Ar–CH), 105.8 (C-2′,6′), 61.0 (OCH₃-4′), 56.4 (OCH₃-3′,5′), 44.6 (NCH₂), 12.6 (CH₃).

(2*E*)-1-(3,4,5-trimethoxyphenyl)-3-(4-acetamidophenyl)prop-2-en-1-one (**11**).

¹H NMR (250 MHz, CDCl₃, 25°C, TMS, ppm): **δ**=7.87 (br s, 1H; NH), 7.76 (d, *J*=15.5 Hz, 1H; Hβ), 7.60–7.56 (m, 2H; Ar–H), 7.52–7.48 (m, 2H; Ar–H), 7.40 (d, *J*=15.5 Hz, 1H; Hα), 7.26 (s, 2H; H-2′,6′), 3.92 (s, 6H; OCH₃-3′,5′), 3.90 (s, 3H; OCH₃-4′), 2.19 (s, 3H; COCH₃); ¹³C NMR (250 MHz, CDCl₃, 25°C, TMS, ppm): **δ**=189.3 (C=O), 168.6 (CONH), 153.1 (C-3′,5′), 144.2 (Cα), 142.4 (C-4″), 140.2 (C-4′), 133.6 (C-1′), 130.6 (C-1), 129.4 (C-2,6), 120.6 (Cβ), 119.7 (C-3,5), 106.1 (C-2′,6′), 60.9 (OCH₃-4′), 56.4 (OCH₃-3′,5′), 24.6 (COCH₃).

(2*E*)-1-(3,4,5-trimethoxyphenyl)-3-(2,5-difluorophenyl)prop-2-en-1-one (**12**).

¹H NMR (600 MHz, CDCl₃, 25°C, TMS, ppm): **δ**=7.86 (d, *J*=15.8 Hz, 1H; Hβ), 7.58 (d, *J*=15.8 Hz, 1H; Hα), 7.38–7.35 (m, 1H; Ar–H), 7.16–7.09 (m, 2H; Ar–H), 7.30 (s, 2H; H-2′,6′), 3.89 (s, 6H; OCH₃-3′,5′), 3.83 (s, 3H; OCH₃-4′); ¹³C NMR (600 MHz, CDCl₃, 25°C, TMS, ppm): **δ**=188.8 (C=O), 157.9 (d, *J*≈250 Hz; C–F), 156.9 (d, *J*≈250 Hz; C–F), 153.2 (C-3′,5′), 142.8 (Cα), 136.1 (Cβ), 133.0 (C-1), 125.3, 125.2 (Ar–CH), 118.2 (Ar–CH), 117.4 (Ar–CH), 115.1 (Ar–CH), 106.2 (C-2′,6′), 61.0 (OCH₃-4′), 56.4 (OCH₃-3′,5′).

(2*E*)-1-(3,4,5-trimethoxyphenyl)-3-(2-fluoro-5-(trifluoromethyl)phenyl)prop-2-en-1-one (**13**).

¹H NMR (600 MHz, DMSO-d₆, 25°C, TMS, ppm): **δ**=8.55 (dd, *J*=6.6, 1.8 Hz, 1H; Ar–H), 8.15 (d, *J*=15.6 Hz, 1H; Hβ), 7.92–7.90 (m, 1H; Ar–H), 7.83 (d, *J*=16.2 Hz, 1H; Hα), 7.59 (t, *J*=9.6 Hz, 1H; Ar–H), 7.45 (s, 2H; H-2′,6′), 3.91 (s, 3H; OCH₃-4′), 3.85 (s, 6H; OCH₃-3′,5′); ¹³C NMR (600 MHz, DMSO-d₆, 25°C, TMS, ppm): **δ**=188.2 (C=O), 153.4 (C-3′,5′), 142.9 (Cα), 133.7 (Cβ), 132.9 (C-1), 129.8, 127.0, 126.5 (Ar–CH), 126.0 (q, *J*≈32 Hz; C–CF₃), 124.0 (q, *J*≈272 Hz; CF₃), 123.9(Ar-CH), 118.0 (Ar–CH), 117.8 (Ar–CH), 107.1 (C-2′,6′), 60.7 (OCH₃-4′), 56.8 (OCH₃-3′,5′).

(2*E*)-1-(3,4,5-trimethoxyphenyl)-3-(2-chloro-5-nitrophenyl)prop-2-en-1-one (**14**)

¹H NMR (600 MHz, CDCl₃, 25°C, TMS, ppm): **δ**=8.63 (d, *J*=2.4 Hz, 1H; Ar–H), 8.22 (dd, *J*=9.0, 3.0 Hz, 1H; Ar–H), 8.16 (d, *J*=15.6 Hz, 1H; Hβ), 7.67 (d, *J*=9.0 Hz, 1H; Ar–H), 7.59 (d, *J*=15.6 Hz, 1H; Hα), 7.32 (s, 2H; H-2′,6′), 4.00 (s, 6H; OCH₃-3′,5′), 3.99 (s, 3H; OCH₃-4′); ¹³C NMR (600 MHz, CDCl₃, 25°C, TMS, ppm): **δ**=188.3 (C=O), 153.3 (C-3′,5′), 146.8 (C–NO₂), 143.1 (Cα), 141.7 (Cβ), 138.0 (C-1), 134.9 (C–Cl), 132.6, 131.3, 126.9, 125.1, 122.6 (Ar–CH), 106.3 (C-2′,6′), 61.1 (OCH₃-4′), 56.5 (OCH₃-3′,5′).

(*2E*)-1-(3,4,5-trimethoxyphenyl)-3-(2-hydroxy-4-nitrophenyl)prop-2-en-1-one (**15**).

¹H NMR (600 MHz, DMSO-d₆, 25°C, TMS, ppm): **δ**=13.00 (br s, 1H; OH), 8.30–8.10 (m, 1H; Ar–H), 7.90 (d, *J*=15.5 Hz, 1H; Hβ), 7.80–7.60 (m, 1H; Ar–H), 7.60 (d, *J*=15.5 Hz, 1H; Hα), 7.20–6.90 (m, 1H; Ar–H), 6.72 (s, 2H; H-2′,6′), 3.88 (s, 6H; OCH₃-3′,5′), 3.80 (s, 3H; OCH₃-4′); ¹³C NMR (600 MHz, DMSO-d₆, 25°C, TMS, ppm): **δ**=188.5 (C=O), 162.5 (C-2″, Ar–OH), 152.4 (C-4″, Ar–NO₂), 152.1 (C-3′,5′), 146.7 (C-4′), 142.3 (Cα), 136.8 (Cβ), 133.1 (C-1″), 127.0 (Ar–CH), 123.6 (Ar–CH), 119.3 (Ar–CH), 139.7 (C-1′), 105.6 (C-2′,6′), 60.8 (OCH₃-4′), 55.9 (OCH₃-3′,5′).

(2*E*)-1-(3,4,5-trimethoxyphenyl)-3-(5-bromo-2-hydroxyphenyl)prop-2-en-1-one (**16**).

¹H NMR (600 MHz, DMSO-d₆, 25°C, TMS, ppm): **δ**=10.62 (s, 1H; OH), 8.14 (d, *J*=2.4 Hz, 1H; Ar–H), 8.00 (d, *J*=15.6 Hz, 1H; Hβ), 7.92 (d, *J*=15.6 Hz, 1H; Hα), 7.43 (dd, *J*=8.4, 2.4 Hz, 1H; Ar–H), 7.43 (s, 2H; H-2′,6′), 6.91 (d, *J*=8.4 Hz, 1H; Ar–H), 3.91 (s, 6H; OCH₃-3′,5′), 3.77 (s, 3H; OCH₃-4′); ¹³C NMR (600 MHz, DMSO-d₆, 25°C, TMS, ppm): **δ**=188.0 (C=O), 156.3 (C–OH), 152.9 (C-3′,5′), 142.0 (Cα), 137.3 (Cβ), 134.2 (C–Br), 133.0 (C-1), 130.2, 123.8, 121.8, 118.3, 110.8 (Ar–CH), 106.3 (C-2′,6′), 60.2 (OCH₃-4′), 56.3 (OCH₃-3′,5′).

(2*E*)-1-(3,4,5-trimethoxyphenyl)-3-(3,5-dimethoxyphenyl)prop-2-en-1-one (**17**).

¹H NMR (600 MHz, CDCl₃, 25°C, TMS): **δ**=8.09 (d, *J*=15.6 Hz, 1H; Hβ), 7.53 (d, *J*=16.2 Hz, 1H; Hα), 7.29 (s, 2H; H-2′,6′), 7.20 (d, *J*=3.0 Hz, 1H; H-4), 6.97 (dd, *J*=9.0, 3.0 Hz, 1H; H-6), 6.91 (d, *J*=9.0 Hz, 1H; H-2), 3.97 (s, 6H; OCH₃-3′,5′), 3.96 (s, 3H; OCH₃-4′), 3.90 (s, 3H; OCH₃-3), 3.85 (s, 3H; OCH₃-5); ¹³C NMR (600 MHz, CDCl₃, 25°C, TMS): **δ**=190.1 (C=O), 153.3 (C-3′,5′), 142.2 (Cα), 140.2 (Cβ), 133.7 (C-1′), 124.6 (C-1), 123.1 (C-4), 116.9 (C-6), 114.0 (C-2), 112.4 (C-3,5), 106.1 (C-2′,6′), 61.0 (OCH₃-4′), 56.4 (OCH₃-3′,5′), 56.1 (OCH₃-3), 55.9 (OCH₃-5).

(2*E*)-1-(3′,4′,5′-trimethoxyphenyl)-3-(3-hydroxy-4-methoxyphenyl)prop-2-en-1-one (**18**).

¹H NMR (CD₂Cl₂, 250 MHz, 25°C, TMS): **δ**=7.69 (d, *J*=15.5 Hz, 1H, H-β), 7.37 (d, *J*=15.5 Hz, 1H, H-α), 7.20 (s, 2H, H-2′, H-6′), 7.15 (dd, *J*=8.1, 2.0 Hz, 1H, H-6), 7.09 (d, *J*=2.0 Hz, 1H, H-2), 6.77 (d, *J*=8.1 Hz, 1H, H-5), 3.89 (s, 3H, CH₃O), 3.86 (s, 6H, 2 × CH₃O), 3.81 (s, 3H, CH₃O); ¹³C NMR (CDCl₃, 250 MHz, 25°C, TMS): **δ**=191.7 (C=O), 158.8 (C-4), 156.8 (C-3′, C-5′), 154.5 (C-4′), 149.4 (C-β, C-3), 135.6 (C-1, C-10), 129.5 (C-α), 119.1 (C-6), 117.1 (C-5), 111.2 (C-2), 107.3 (C-2′, C-6′), 61.3 (CH₃O), 56.9 (2 × CH₃O), 55.8 (CH₃O).

(2*E*)-1-(3′,4′,5′-trimethoxyphenyl)-3-(3,4-dimethoxyphenyl)prop-2-en-1-one (**19**).

¹H NMR (600 MHz, CDCl₃, 25°C, TMS): **δ**=7.74 (d, *J*=15.5 Hz, 1H, H-β), 7.31 (d, *J*=16.0 Hz, 1H, H-α), 7.24 (dd, *J*=8.0, 2.0 Hz, 1H, H-6), 7.24 (dd, *J*=8.0, 2.0 Hz, 1H, H-2′), 7.24 (dd, *J*=8.0, 2.0 Hz, 1H, H-6′), 7.13 (d, *J*=2.0 Hz, 1H, H-2), 6.88 (d, *J*=8.5 Hz, 1H, H-5), 3.93 (s, 3H, OCH₃-4), 3.92 (s, 6H, OCH₃-3′, OCH₃-5′), 3.91 (s, 6H, OCH₃-3, OCH₃-4′); ¹³C NMR (600 MHz, CDCl₃, 25°C, TMS): **δ**=189.5 (C=O), 153.2 (C-3′, C-5′), 151.5 (C-4), 149.3 (C-3), 145.1 (C-β), 142.4 (C-α), 133.9 (C-1), 127.9 (C-6), 123.0 (C-1′), 119.9 (C-5), 111.2 (C-2), 110.6 (C-6′), 106.2 (C-2′, C-6′), 61.1 (OCH₃-4′), 56.5 (OCH₃-3′, C-5′), 56.1 (OCH₃-3, C-4).

(2*E*)-1-(3′,4′,5′-trimethoxyphenyl)-3-(3,4-methylenedioxyphenyl)prop-2-en-1-one (**20**).

¹H NMR (250 MHz, CDCl₃, 25°C, TMS): **δ**=7.74 (d, *J*=15.2 Hz, 1H, H-β), 7.31 (d, *J*=15.6 Hz, 1H, H-α), 7.16 (d, *J*=1.6 Hz, 1H, H-2), 7.13 (dd, *J*=8.0, 1.6 Hz, 1H, H-6), 6.85 (d, *J*=8.0 Hz, 1H, H-5), 6.03 (s, 2H, O–CH₂–O), 3.95 (s, 6H, OCH₃-3′, OCH₃-5′), 3.93 (s, 3H, OCH₃-4′); ¹³C NMR (250 MHz, CDCl₃, 25°C, TMS): **δ**=189.5 (C=O), 153.6 (C-3′, C-5′), 150.4 (C-3), 148.8 (C-4), 145.0 (C-β), 134.1 (C-1), 129.8 (C-α), 125.6 (C-6), 120.2 (C-5), 109.1 (C-2), 107.1 (C-2′, C-6′), 106.6 (C-1′), 102.1 (O–CH₂–O), 61.4 (OCH₃-4′), 56.8 (OCH₃-3′, C-5′).

(2*E*)-1-(3,4,5-trimethoxyphenyl)-3-(1H-indol-5-yl)prop-2-en-1-one (**21**).

¹H NMR (600 MHz, DMSO-d₆, 25°C, TMS): **δ**=11.39 (s, 1H, NH), 8.09 (s, 1H, Ar–H), 7.90 (d, *J*=15.6 Hz, 1H, H-1″), 7.88 (d, *J*=15.6 Hz, 1H, H-2″), 7.75 (dd, *J*=8.4, 1.2 Hz, 1H, Ar–H), 7.48 (d, *J*=8.4 Hz, 1H, Ar–H), 7.45 (s, 2H, H-2′, H-6′), 7.44 (d, *J*=3.0 Hz, 1H, Ar–H), 6.53 (d, *J*=2.4 Hz, 1H, Ar–H), 3.93 (s, 6H, OCH₃-3′, OCH₃-5′), 3.78 (s, 3H, OCH₃-4′); ¹³C NMR (600 MHz, DMSO-d₆, 25°C, TMS): **δ**=187.67 (C=O), 152.86 (C-3′, C-5′), 146.49 (C-4′), 141.57 (C-β), 137.40 (C-α), 133.51 (Ar–C), 127.92 (Ar–C), 126.73 (Ar–C), 125.88 (Ar–C), 123.10 (Ar–C), 121.75 (Ar–C), 118.09 (Ar–C), 111.91 (Ar–C), 105.88 (C-2′, C-6′), 102.12 (C-1′), 60.15 (OCH₃-4′), 56.15 (OCH₃-3′, C-5′).

(2*E*)-1-(3,4,5-trimethoxyphenyl)-3-(2,4,6-trimethylphenyl)prop-2-en-1-one (**22**).

¹H NMR (600 MHz, CDCl₃, 25°C, TMS): **δ**=7.95 (d, *J*=16.2 Hz, 1H, H-β), 7.26 (s, 2H, H-2′, H-6′), 7.12 (d, *J*=16.2 Hz, 1H, H-α), 3.95 (s, 2H, Ar–H), 3.94 (s, 3H, OCH₃-4′), 3.93 (s, 6H, OCH₃-3′, OCH₃-5′), 2.39 (s, 6H, CH₃-2, CH₃-6), 2.31 (s, 3H, CH₃-4); ¹³C NMR (600 MHz, CDCl₃, 25°C, TMS): **δ**=189.24 (C=O), 153.16 (C-3′, C-5′), 143.21 (C-β), 142.37 (C-α), 138.62 (C-2, C-6), 137.06 (C-4), 133.42 (C-1′), 131.80 (C-1), 129.28 (Ar–CH), 127.36 (Ar–CH), 105.99 (C-2′, C-6′), 61.03 (OCH₃-4′), 56.32 (OCH₃-3′, C-5′), 21.23 (CH₃-2, CH₃-6), 21.13 (CH₃-4).

(2*E*)-1-(3,4,5-trimethoxyphenyl)-3-(2,4,6-trimethoxyphenyl)prop-2-en-1-one (**23**).

¹H NMR (250 MHz, CDCl₃, 25°C, TMS): **δ**=8.23 (d, *J*=15.6 Hz, 1H, H-β), 7.82 (d, *J*=16.0 Hz, 1H, H-α), 7.28 (s, 2H, H-2′, H-6′), 6.14 (s, 2H, H-3, H-5), 3.94 (s, 6H, OCH₃-3′, OCH₃-5′), 3.93 (s, 3H, OCH₃-4′), 3.90 (s, 6H, OCH₃-2, OCH₃-6), 3.86 (s, 3H, OCH₃-4); ¹³C NMR (250 MHz, CDCl₃, 25°C, TMS): **δ**=190.9 (C=O), 162.9 (C-2, C-6), 161.4 (C-4), 152.7 (C-3′, C-5′), 141.6 (C-β), 135.8 (C-α), 134.4 (C-1′), 121.8 (C-1), 106.4 (C-2′, C-6′), 105.9 (C-3, C-5), 90.4 (C-1″), 60.7 (OCH₃-4′), 56.0 (OCH₃-3′, C-5′), 55.6 (OCH₃-2, C-6), 55.2 (OCH₃-4).

(2*E*)-1-(3,4,5-trimethoxyphenyl)-3-(3,5-dimethoxyphenyl)prop-2-en-1-one (**24**).

¹H NMR (250 MHz, CD₃OD, 25°C, TMS): **δ**=7.74 (d, *J*=15.5 Hz, 1H, H-β), 7.59 (d, *J*=15.5 Hz, 1H, H-α), 7.40 (s, 2H, H-2′, H-6′), 7.08 (s, 2H, H-2, H-6), 4.85 (s, 1H, Ar–H), 3.94 (s, 6H, OCH₃-3′, OCH₃-5′), 3.93 (s, 6H, OCH₃-3, OCH₃-5), 3.88 (s, 3H, OCH₃-4′); ¹³C NMR (250 MHz, CD₃OD, 25°C, TMS): **δ**=191.27 (C=O), 154.57 (C-3′, C-5′), 149.54 (C-3, C-5), 147.45 (C-4′), 143.82 (C-β), 140.20 (C-α), 135.17 (C-1′), 127.30 (C-1), 120.08 (Ar–CH), 107.82 (C-2′, C-6′), 107.54 (C-2, C-6), 61.25 (OCH₃-4′), 56.99 (OCH₃-3′, C-5′), 56.93 (OCH₃-3, C-5).

(2*E*)-1-(3,4,5-trimethoxyphenyl)-3-(3,4,5-trimethoxyphenyl)prop-2-en-1-one (**25**).

¹H NMR (250 MHz, CDCl₃, 25°C, TMS): **δ**=7.70 (d, *J*=15.6 Hz, 1H, H-β), 7.32 (d, *J*=15.6 Hz, 1H, H-α), 7.25 (s, 2H, H-2′, H-6′), 6.85 (s, 2H, H-2, H-6), 3.94 (s, 6H, OCH₃-3′, OCH₃-5′), 3.93 (s, 3H, OCH₃-4′), 3.91 (s, 6H, OCH₃-3, OCH₃-5), 3.89 (s, 3H, OCH₃-4); ¹³C NMR (250 MHz, CDCl₃, 25°C, TMS): **δ**=189.5 (C=O), 153.7 (C-3′, C-5′), 153.3 (C-3, C-5), 145.0 (C-β), 133.7 (C-α), 130.5 (C-1′, C-1), 121.5 (Ar–CH), 106.5 (C-2′, C-6′), 106.0 (C-2, C-6), 61.1 (OCH₃-4′), 56.6 (OCH₃-3′, C-5′), 56.4 (OCH₃-3, C-5).
